# Effects of fNIRS Processing Methods on Prefrontal Hemodynamics During Single and Dual Task Walking in Older Adults

**DOI:** 10.1093/geroni/igaa057.922

**Published:** 2020-12-16

**Authors:** Meltem Izzetoglu, Roee Holtzer

**Affiliations:** 1 Villanova University, Villanova, Pennsylvania, United States; 2 Yeshiva University, Bronx, New York, United States

## Abstract

functional near infrared spectroscopy (fNIRS) has been increasingly used to assess changes in the hemodynamic response during active walking in aging and disease populations. Key findings revealed that HbO2 in the prefrontal cortex (PFC) increased from single-task-walk (STW) to dual-task-walk (DTW) due to the greater cognitive demands inherent in the latter condition. However, previous studies utilized a limited and inconsistent number of algorithms and filters to remove artifacts from fNIRS-derived brain activation data. Critically, there is no gold standard for artifact removal at the present time, which reduces replicability and generalizability. To address this critical limitation, we have reanalyzed a large dataset of older adults (n=83) who underwent our walking protocol by using different hemodynamic conversion parameters (molar extinction coefficients and age and wavelength dependent differential pathlength factors) and applying different filters having various cut-off frequencies for artifact removal. On the extracted hemodynamic responses, namely oxygenated-hemoglobin (HbO2) and deoxygenated-hemoglobin (Hb), linear mixed effect model results indicated that task effects showed similar significant increases in HbO2 from STW to DTW (range of effect sizes was 0.59 to 0.64) and as well as the expected decline in Hb from STW to DTW (range of effect sizes was 0.18 to 0.32) irrespective of the methods used. In addition, the intraclass correlations suggested excellent reliability across methods (HbO2 range = 0.982 to 0.996; Hb range = 0.883 to 0.984). In conclusion, these findings provide strong support to previously published articles but also highlight the need to establish a gold standard for fNIRS processing.

